# Predicting post‐stroke dementia using sex‐specific risk factors: Development of an interpretable clinical scoring tool

**DOI:** 10.1002/dad2.70336

**Published:** 2026-04-27

**Authors:** Chenyin Chu, Yihan Wang, Liwei Ma, Liang Jin, Yijun Pan

**Affiliations:** ^1^ School of Translational Medicine Monash University Melbourne Victoria Australia; ^2^ School of Health and Biomedical Sciences The Royal Melbourne Institute of Technology Bundoora Victoria Australia

**Keywords:** AutoScore algorithm, post‐stroke dementia, risk stratification, ShapelyVIC, vascular dementia

## Abstract

**INTRODUCTION:**

Post‐stroke cognitive impairment/dementia are disabling outcomes, yet interpretable prognostic tools remain limited. We aim to develop prediction tools accounting for sex‐specific risk profiles.

**METHODS:**

We analyzed 766 stroke patients (mean age, 79.1 years; 377 men, 389 women). Shapley Variable Importance Cloud (ShapleyVIC) identified stable predictors, which were fed to the AutoScore framework to construct the Monash Stroke Dementia Score (MSDS). Model performance was evaluated using an area under the receiver operating characteristic curve (AUC), along with sensitivity, specificity, positive predictive value, and negative predictive value.

**RESULTS:**

The MSDS achieved an AUC of 0.81 (95% confidence interval [CI], 0.78–0.84), with a sensitivity of 0.78 and specificity of 0.77. Although the overall risk of post‐stroke dementia was similar between men and women, sex‐specific models demonstrated improved discrimination and distinct risk profiles.

**DISCUSSION:**

The MSDS provides a robust, interpretable tool for individualized prediction of post‐stroke cognitive impairment/dementia, with distinct sex‐specific risk patterns.

## Highlights

- Post‐stroke dementia is common, and early predictions are critical for patient care.
- The Monash Stroke Dementia Score (MSDS) provides a simple point‐based tool.
- Sex‐specific models capture distinct male and female risk factor profiles.
- The Shapley Variable Importance Cloud (ShapleyVIC) enhances MSDS efficiency and robustness, achieving high accuracy.
- Future validation in diverse cohorts is required for broader clinical adoption.

## 1 BACKGROUND

Stroke is the second leading cause of death and a major cause of disability worldwide, with more than 12 million new cases annually and nearly 7 million deaths each year.^1^ Beyond motor and physical sequelae, stroke survivors are at a markedly elevated risk of cognitive impairment and dementia, conditions that substantially worsen prognosis, functional independence, and quality of life.^2^ Approximately one‐third of patients develop post‐stroke cognitive impairment, and up to 20% progress to dementia within 5 years, making it one of the most disabling long‐term outcomes of stroke.^3^, ^4^

**RESEARCH IN CONTEXT**

**1. Systematic review**: A targeted review of PubMed, Web of Science, and Google Scholar revealed that while post‐stroke cognitive impairment and dementia have been extensively studied epidemiologically, existing prediction models are scarce, often lack of interpretability, and rarely incorporate sex‐specific analyses. No parsimonious, validated clinical score currently exists to support individualized post‐stroke dementia risk stratification.
**2. Interpretation**: Using the National Alzheimer's Coordinating Center (NACC) dataset, we developed the Monash Stroke Dementia Score (MSDS), an interpretable risk score constructed through the AutoScore framework enhanced by Shapley Variable Importance Cloud (ShapleyVIC) for stable feature selection. The MSDS demonstrated a strong predictive performance (area under the receiver operating characteristic curve [AUC] = 0.81) and was further refined into sex‐specific models, which uncovered distinct vascular, social factor, clinical assessment, and neuropsychiatric predictors in men and women.
**3. Future directions**: Future studies should validate the MSDS in prospective, population‐based cohorts, incorporate imaging and biomarker data, and assess its utility in guiding individualized post‐stroke care.

A reliable clinical tool for predicting the risk of post‐stroke dementia could enable clinicians to identify high‐risk patients early. However, prediction tools for post‐stroke dementia remain scarce and there is no established approach to predict the occurrence of post‐stroke dementia in the clinic setting. Machine learning has emerged as a promising approach, offering enhanced predictive accuracy and the ability to integrate high‐dimensional data.^5^, ^6^ However, machine learning models often function as “black boxes” and lack clinical interpretability thereby limiting their applicability in routine clinical practice.^7^, ^8^ On the other hand, sex differences represent an important yet underexplored dimension in post‐stroke cognitive outcomes. Women and men may differ in vascular risk burden, cognitive resilience, and long‐term prognosis, with recent studies reporting distinct risk profiles and rates of progression to dementia.^9^, ^10^ Nevertheless, few predictive models have incorporated sex‐specific analyses or developed tailored scoring systems, despite growing calls for personalized approaches in cerebrovascular and dementia care.

To address this, we developed and validated the Monash Stroke Dementia Score (MSDS), an interpretable model for predicting post‐stroke dementia. Using data from the National Alzheimer's Coordinating Center (NACC) cohort, we applied the Shapley Variable Importance Cloud (ShapleyVIC) to identify risk predictors and integrated these into the AutoScore framework to generate a parsimonious, point‐based score. Sex‐specific models were developed to characterize differential risk profiles in men and women. All the machine learning findings were verified using epidemiology analyses to enhance stability, reproducibility, and clinical relevance.^11^, ^12^

## 2 METHODS

### 2.1 Data sources and ethics

This study used data from the NACC,^13^ which includes detailed information on cognitive status, demographics, medical and family history, and clinical assessments of cognitive, motor, functional, and neuropsychiatric health, collected prospectively by trained clinicians at annual visits. Data were accessed through the NACC website (https://naccdata.org/). This study also used data from the Religious Orders Study and Memory and Aging Project (ROSMAP).^14^, ^15^, ^16^ Both studies recruit participants without known dementia at enrollment and follow them prospectively with detailed clinical, cognitive, and neuropathological assessments. The ROSMAP study was approved by the Institutional Review Board of Rush University Medical Center, and all participants provided written informed consent. Data were obtained from the Rush Alzheimer's Disease Center at https://www.radc.rush.edu. This study involved secondary analysis of deidentified data, with ethical approval granted by Monash University (Project ID 48297).

### 2.2 Study design

This prognostic study used the ShapleyVIC method to identify risk factors for post‐stroke dementia, accounting for variability in variable importance across models.^17^ Identified features were examined in epidemiologic analyses and incorporated into the MSDS with the AutoScore framework.^18^ The first documented stroke was defined as the index event, and all analyses were anchored at the time of the first stroke, with longitudinal follow‐up extending to the participant's last recorded visit in the cohort.

### 2.3 Participant and feature pre‐processing

Of the 51,836 participants in the NACC cohort, 1134 individuals with a documented history of stroke were included, with the analyzed features summarized in eTable 1. After restricting the sample to participants with complete data on all variables of interest, the final analytic cohort comprised 766 individuals (389 women and 377 men). Participants without a diagnosis of stroke‐related dementia at the last recorded follow‐up, including those who were cognitively unimpaired and those with mild cognitive impairment, were classified as the non‐dementia group. All predictor variables, including Mini‐Mental State Examination (MMSE), Clinical Dementia Rating scale Sum of Boxes (CDR‐SB), Geriatric Depression Scale (GDS), and neurological findings, were assessed at the time of the first recorded stroke and follow‐up visits.

In the NACC dataset, post‐stroke dementia was operationalized as a new clinical diagnosis of dementia occurring after the first documented stroke, with explicit clinician documentation indicating that stroke was judged to have contributed to the participant's cognitive impairment, based on standardized diagnostic and attribution variables. Participants with any documented diagnosis of dementia prior to the first stroke were excluded to ensure that only incident stroke‐related dementia cases were included.

### 2.4 MSDS model development and evaluation

The MSDS predicts the occurrence of stroke‐related dementia during follow‐up as a binary outcome, expressed as a corresponding risk probability. The final analytic NACC dataset was randomly divided at the individual level into a training/validation set (70%) and a test set (30%). Model development followed the AutoScore framework,^18^ with feature importance ranking performed using the ShapleyVIC.^17^ Continuous predictors were transformed using algorithm‐derived cut‐points to reduce the influence of nonlinearity and outliers on model performance,^19^ followed by clinical refinement to ensure consistency with accepted clinical norms and clinical guidelines. Score derivation was conducted using logistic regression, with coefficients normalized and rescaled to generate an integer‐based scoring system. Parsimony analysis was performed by sequentially adding predictors and evaluating discrimination using a 10‐fold cross‐validation in the training/validation set, identifying a minimal feature set that balanced model simplicity and performance. The final model was evaluated on the test set. The overall workflow is illustrated in **eFigure** **1**, and detailed technical specifications are provided in the eMethods.

Model performance was assessed using the area under the receiver operating characteristic curve (AUC), sensitivity, specificity, positive predictive value (PPV), and negative predictive value (NPV), with 95% bootstrap confidence intervals. The MSDS was compared with baseline models, including the original AutoScore framework and logistic regression models incorporating random forest (RF) ‐based feature selection with SHapley Additive exPlanations (SHAP), with differences in AUC evaluated using the Wilcoxon signed‐rank test.

External validation was performed using an independent cohort from the ROSMAP, comprising 262 participants with a history of stroke. Data preprocessing and inclusion criteria were aligned with those used in the NACC cohort. The MSDS was applied to the ROSMAP cohort without model refitting, and performance was evaluated using the same metrics as in the internal test set.

### 2.5 Sex‐specific model development and epidemiologic analyses

Given prior evidence that post‐stroke cognitive impairment differs by sex,^9^, ^10^, ^20^ sex‐specific MSDS models were developed for men (*n* = 377) and women (*n* = 389). These models, referred to as the male‐MSDS and female‐MSDS, followed the same modeling structure as the overall MSDS. ShapleyVIC was applied within each sex‐specific subgroup to identify predictors that contributed most strongly to post‐stroke cognitive impairment and dementia.

To support the robustness and clinical plausibility of the identified predictors, complementary epidemiologic analyses were performed using multivariable logistic regression. These analyses evaluated associations between candidate risk factors and post‐stroke cognitive impairment while adjusting for relevant demographic covariates. Details about analysis are provided in the eMethods.

## 3 RESULTS

### 3.1 Baseline characteristics

The NACC cohort comprised 766 participants (mean [SD] age, 79.1 [8.6] years; 377 men and 389 women) (Table 1). Men and women were of similar age (78.9 [8.1] vs 79.3 [9.2] years; *p* = 0.52). Men reported more years of education (15.8 [8.4] vs 14.1 [8.3]; *p* = 0.004) and a longer smoking history (16.1 [18.2] vs 11.7 [18.6] years; *p* < 0.001). The mean follow‐up duration was slightly longer in women than in men (2.2 [2.1] vs 1.9 [1.8] years; *p* = 0.03). Cognitive function did not differ significantly by sex, with mean CDR‐SB scores of 3.8 (4.0) and 3.6 (4.0) (*p* = 0.42) and MMSE scores of 24.0 (5.5) and 24.6 (5.3) (*p* = 0.16) in men and women, respectively. Post‐stroke dementia was diagnosed in 37.9% of men and 36.7% of women (*p* = 0.51).

**TABLE 1:** Baseline characteristics.

Features	ROSMAP (*n* = 262)	NACC (*n* = 766)	Male (*n* = 377)	Female (*n* = 389)	*p*‐Value
Age, mean (SD), years	82.0 (7.3)	79.1 (8.6)	78.9 (8.1)	79.3 (9.2)	0.52
Education, mean (SD), years	15.4 (3.5)	14.9 (8.4)	15.8 (8.4)	14.1 (8.3)	**0.004**
Diastolic blood pressure, mean (SD), mmHg	72.9 (11.8)	74.3 (10.5)	74.5 (10.4)	74.1 (10.7)	0.57
Systolic blood pressure, mean (SD), mmHg	133.4 (16.9)	136.1 (19.1)	135.9 (19.1)	136.2 (19.1)	0.82
Heart rate, mean (SD), beats/min	n/a^	68.5 (11.4)	66.6 (11.2)	70.4 (11.2)	**<0.001**
Follow‐up, mean (SD), years	4.5 (4.2)	2.1 (2.0)	1.9 (1.8)	2.2 (2.1)	**0.03**
CDR‐SB, mean (SD)	3.2 (3.7)	3.7 (4.0)	3.8 (4.0)	3.6 (4.0)	0.42
MMSE, mean (SD)	25.2 (5.6)	24.3 (5.4)	24.0 (5.5)	24.6 (5.3)	0.16
Smoking, mean (SD), years	n/a^	13.8 (18.5)	16.1 (18.2)	11.7 (18.6)	**<0.001**
GDS score, mean (SD)	2.8 (2.7)	2.7 (2.8)	2.6 (2.6)	2.8 (2.9)	0.29
Stroke contributes to dementia, No. (%)	185 (70.6) / 77 (19.4)	570 (74.4) / 196 (25.6)	285 (75.6) / 92 (24.4)	285 (73.3) / 104 (26.7)	0.51

Abbreviations: CDR‐SB, Clinical Dementia Rating–Sum of Boxes; GDS, Geriatric Depression Scale; MMSE, Mini‐Mental State Examination; NACC, National Alzheimer's Coordinating Center; ROSMAP, Religious Orders Study and Memory and Aging Project; SD, standard deviation.

* Statistically significant difference between men and women (*p*< 0.05).

^These data are not recorded in the ROSMAP study but not used as selected predictors.

In the external validation cohort, the ROSMAP sample included 262 participants with a documented history of stroke (mean [SD] age, 82.0 [7.3] years). Compared with the NACC cohort, ROSMAP participants were older and had a longer mean follow‐up duration (4.5 [4.2] vs 2.1 [2.0] years). Baseline cognitive performance was comparable between cohorts, with mean CDR‐SB and MMSE scores of 3.2 (3.7) and 25.2 (5.6), respectively, in the ROSMAP cohort (Table 1).

### 3.2 Outcomes of MSDS model construction and validation

**FIGURE 1:**
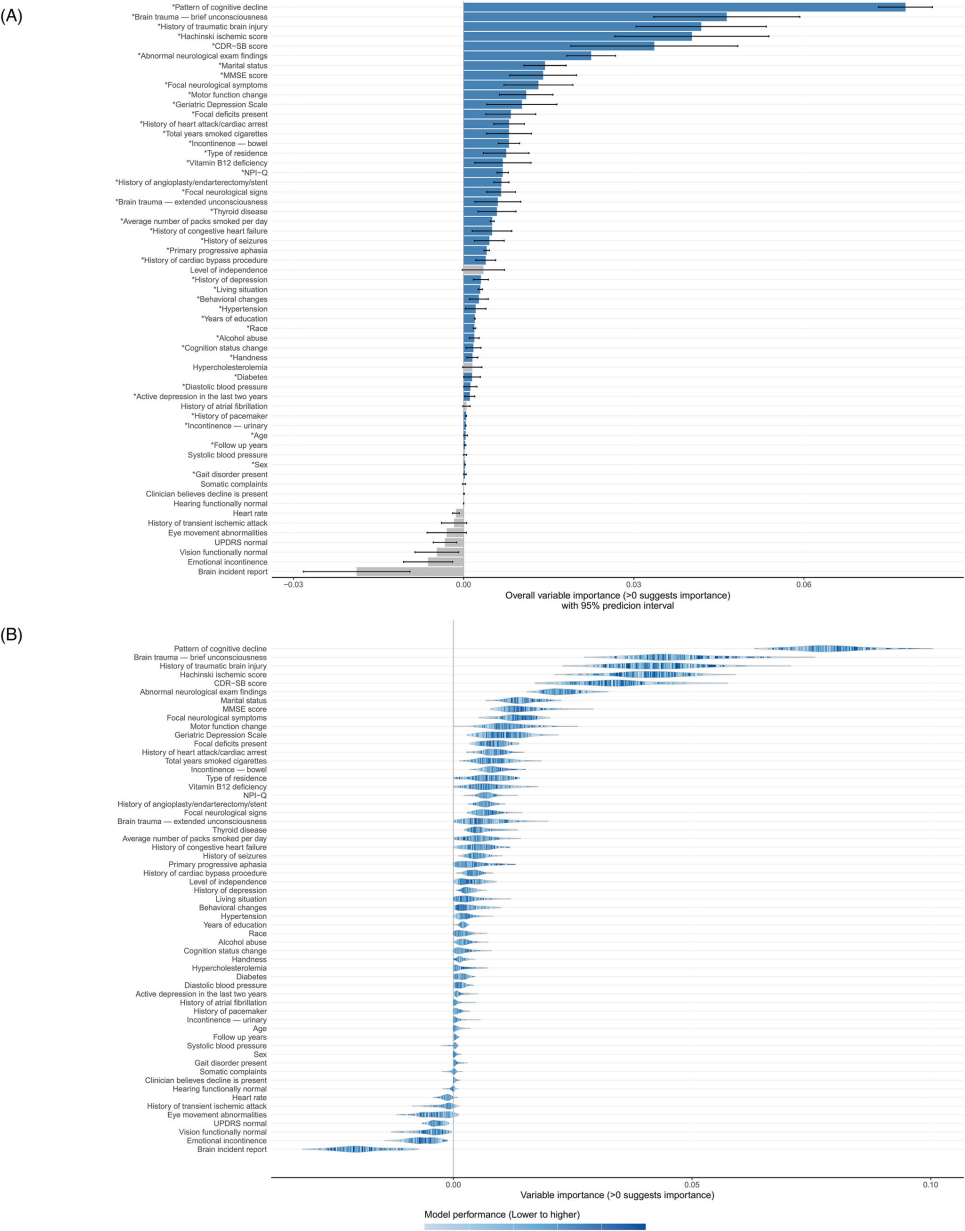
ShapleyVIC values with 95% PIs and distribution. (A) Average ShapleyVIC values with 95% PIs, with nonpositive values shown as gray bars to indicate negligible importance. (B) The distribution of ShapleyVIC values across nearly optimal models to demonstrate their association with model performance. PI, predictive interval; ShapleyVIC, Shapley Variable Importance Cloud.

ShapleyVIC analyses (Figures 1A,B) identified pattern of cognitive decline as the most important predictor (ShapleyVIC = 0.08), supported by narrow prediction intervals and consistently high values across models. Brain trauma with brief unconsciousness was of the second highest importance (0.05), followed by traumatic brain injury (0.04), Hachinski Ischemic Scale (0.04), and baseline CDR‐SB score (0.03). Moderate contributions were observed for abnormal neurological exam findings, marital status, baseline MMSE, focal neurological symptoms, changes in motor function, GDS, and central nervous system disorder (0.01–0.02). In contrast, features such as independent living status and hypercholesterolemia had nonpositive ShapleyVIC values, indicating minimal influence on prediction, and were excluded from subsequent analyses. Across the 250 nearly optimal models, variable importance was averaged to generate an ensemble ranking, from which 45 variables with significant overall contributions were retained for further development.

**FIGURE 2:**
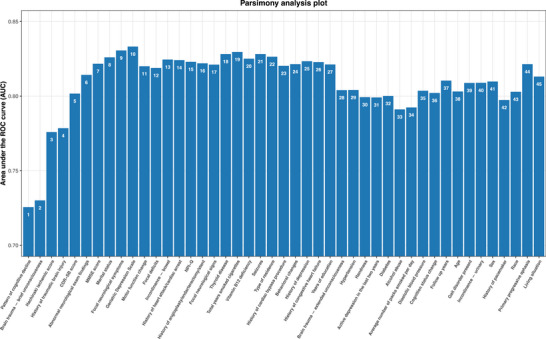
Parsimony plot for MSDS. This plot illustrates the relationship between the number of predictors included in the MSDS model and its performance, measured by the mean area under the receiver operating characteristic curve across a 10‐fold cross‐validation. Numbers above the bars indicate the cumulative count of predictors incorporated at each step. Taller bars represent superior model performance. MSDS, Monash Stroke Dementia Score.

After the ShapleyVIC importance ranking, a parsimony analysis was performed to evaluate incremental gains in model performance (Figure 2). Model performance, measured by the AUC, improved from 0.72 to 0.78 with the inclusion of the first three features. Addition of the fourth feature provided only modest improvement, whereas inclusion of the fifth through tenth features increased the AUC steadily from 0.78 to 0.83. Beyond 10 features, no evident gains in AUC were observed. Accordingly, the number of features was set at 10 (hyperparameter) to balance predictive performance and model complexity.

When identifying the features included in the MSDS, a logistic regression model was applied to assign scores for each category or interval of the selected predictors. To enhance clinical interpretability, intervals were refined according to established clinical norms. The Hachinski Ischemic Scale was categorized as < 4, 5–6, and ≥7.^21^ The MMSE was categorized as < 10, 10–17, 18–23, and ≥24,^22^ whereas the CDR‐SB score was categorized as < 1, 1–4, and ≥4.5.^23^ The GDS was categorized as < 5, 5–10, and > 10.^24^ The revised scores for each feature after fine‐tuning are presented in Table 2A.

**TABLE 2A:** Score table for MSDS after fine‐tuning.

Feature	Interval	Score
Pattern of cognitive decline	Stepwise or gradual	0
	Abrupt	28
Brain trauma brief unconsciousness	Absent	0
	Remote/inactive	4
	Recent/active	8
Hachinski Ischemic Scale*	<4	0
	[4,6)	6
	≥6	8
Traumatic brain injury	No	0
	Yes	4
Clinical Dementia Rating scale Sum of Boxes (CDR‐SB)*	<1	0
	[1,4)	5
	[4,9)	11
	≥9	12
Abnormal neurological findings	No	0
	Yes	6
Mini‐Mental State Examination (MMSE) score*	<10	12
	[10,17)	8
	[17,24)	6
	≥24	0
Married status	Married	0
	Widowed	0
	Never married	3
Focal neurological symptoms	No	0
	Yes	4
Geriatric Depression Scale (GDS)‐15 items*	<4	0
	[4,8)	2
	[8,11)	6
	≥11	13

* Fine‐tuning based on clinical norm. Score threshold for classification is 52.

**FIGURE 3:**
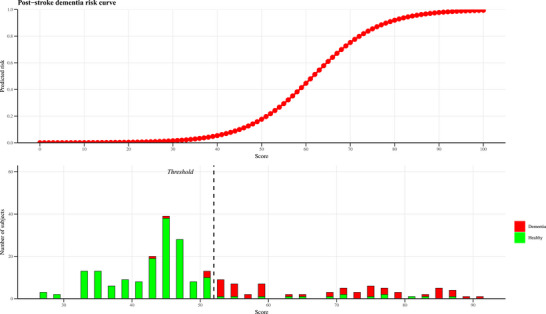
Post‐stroke dementia risk curve and score distribution. The top panel presents the risk curve, depicting the predicted probability of developing dementia (*y*‐axis) across scores ranging from 0 to 100 (*x*‐axis). The bottom panel displays the score distribution as histograms for healthy participants (green bars) and participants with dementia (red bars). The dashed line indicates the binary classification threshold, with scores ≤52 classified as dementia free.

After fine‐tuning, the MSDS model was evaluated on the test set, achieving an AUC of 0.81 (95% CI: 0.78–0.84). Additional performance metrics, with 95% CI derived from 100 bootstrap samples, were as follows: sensitivity of 0.78 (95% CI: 0.74–0.82), specificity of 0.77 (95% CI: 0.71–0.84), PPV of 0.70 (95% CI: 0.69–0.72), and NPV of 0.81 (95% CI: 0.79–0.84). We subsequently mapped the scores from the MSDS model to risk. A risk plot (Figure 3) illustrates the relationship between scores and the probability of post‐stroke dementia, revealing a clear trend of increasing probability with higher scores. These findings underscore the ability of MSDS to effectively predict post‐stroke dementia risk with the chosen features.

### 3.3 Comparison of MSDS with baseline models and external validation of MSDS

The RF‐based feature importance ranking and parsimony analysis are presented in eFigure 2. Like the MSDS model, the Hachinski Ischemic Scale score and pattern of cognitive decline consistently emerged as the top predictors, although ranking of the subsequent features differed. The AUC improved from 0.75 to 0.82 as the number of features increased from 1 to 14, with no further gains beyond that point. Consequently, the original AutoScore framework required 14 features to reach optimal performance, with test set metrics summarized in eTable 2. Notably, despite utilizing more features, the RF‐based model demonstrated an inferior performance compared to the ShapleyVIC‐based selection, which achieved a better discriminatory power with fewer variables. These findings suggest that ShapleyVIC not only improves predictive accuracy but also facilitates a more parsimonious model. Moreover, compared with the SHAP‐based logistic‐RF model (eTable 2, eFigure 3), the MSDS also demonstrated superior performance and greater interpretability.

External validation of the MSDS demonstrated performance consistent with that observed in the internal test set, with an AUC of 0.78 (95% CI, 0.75–0.80), sensitivity of 0.74 (95% CI, 0.72–0.77), specificity of 0.78 (95% CI, 0.74–0.82), PPV of 0.70 (95% CI, 0.68–0.73), and NPV of 0.79 (95% CI, 0.75–0.81).

### 3.4 Sex‐specific MSDS model and risk factor

In sex‐stratified analyses, partially overlapping yet distinct risk profiles emerged. Among men (eFigure 4A,B), the leading predictors were pattern of cognitive decline, traumatic brain injury, Hachinski Ischemic Scale score, and primary progressive aphasia, with additional contributions from focal neurological deficits, focal signs, angioplasty or stent history, marital status, and abnormal neurological findings. Among women (eFigure 4C,D), the strongest predictors were Hachinski Ischemic Scale score, focal neurological symptoms, pattern of cognitive decline, and smoking, with further contributions from marital status, congestive heart failure, cognition status changes, seizure history, living situation, angioplasty or stent history, independence in daily living, and focal neurological deficits. The sex‐specific parsimony analyses (eFigure 5A,B) identified 9 optimal features for men and 15 for women. Corresponding sex‐specific score tables are shown as Table 2B and performance metrics for the sex‐specific MSDS models are summarized in eTable 2. These findings suggest that predictors of post‐stroke dementia differ by sex, underscoring the potential value of tailored risk models.

**TABLE 2B:** Score table for sex‐specific MSDS after fine‐tuning.

Feature	Interval	Score	Score
Hachinski Ischemic Scale*	<4	0	0
	[4,6)	4	1
	≥6	12	9
Focal neurological symptoms	No	0	
	Yes	6	
Pattern of cognitive decline	Stepwise or gradual	0	0
	Abrupt	18	29
Clinician judgment of cognitive function change	<4	0	
	≥4	2	
No. of packs smoked per day	No smoking	0	
	1 to less than 1 pack	1	
	More 1 pack	2	
Congestive heart failure	Absent	0	
	Recent/Active	7	
	Remote/Inactive	14	
Seizures	Absent	0	
	Recent/Active	11	
	Remote/Inactive	7	
Primary progressive aphasia	No	0	0
	Yes	5	17
Married status	Married	3	0
	Widowed	2	4
	Never married	0	6
Focal deficits	No	0	0
	Yes	4	7
Heart attack/cardiac arrest	No	0	
	Yes	2	
Focal neurological signs	No	0	0
	Yes	2	4
Living independent	No	0	
	Yes	10	
Abnormal neurological findings	No	0	0
	Yes	5	6
Clinician judgment of cognition decline	No	0	
	Yes	5	
Traumatic brain injury	No		0
	Yes		4
Angioplasty/endarterectomy/stent	Absent		0
	Recent/Active		4
	Remote/Inactive		11

* Fine‐tuning based on clinical norm. Female (purple) score threshold is 48 and male (blue) score threshold is 58.

### 3.5 Epidemiology findings

**FIGURE 4:**
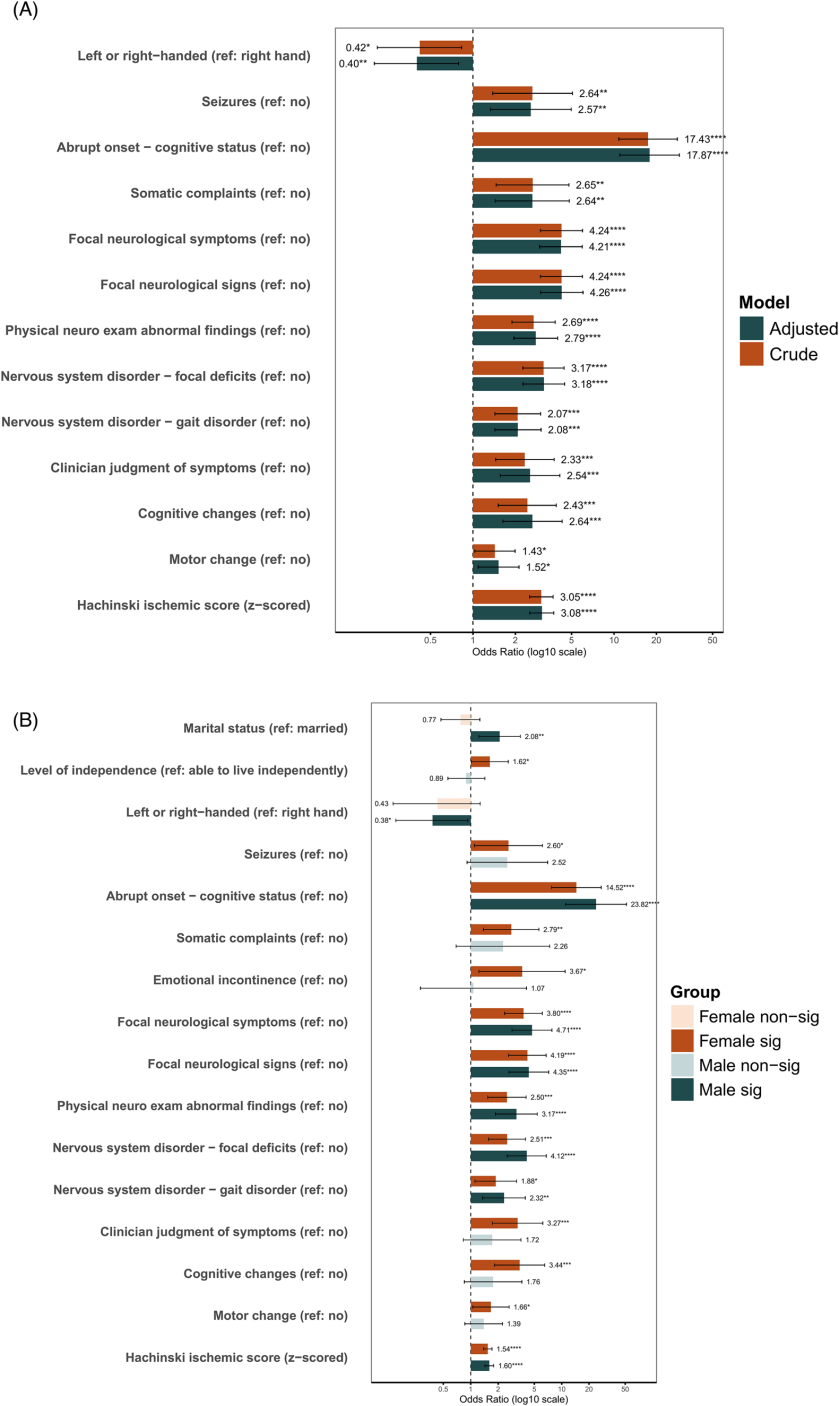
Epidemiologic associations with post‐stroke dementia. (A) Adjusted odds ratios for significant risk factors associated with post‐stroke dementia in the overall cohort. (B) Sex‐specific associations, highlighting differential effects of neurological and behavioral predictors between men and women. All models are adjusted for age, education, and race; error bars indicate 95% confidence intervals.

Epidemiologic analyses were conducted to identify risk factors associated with post‐stroke dementia, with full statistical results provided in eTable 3.1 and eTable 3.2. After multivariable adjustment, several clinical and neurological features including seizure history, pattern of cognitive impairment, abnormal neurological findings, clinician judgment of cognitive or functional decline, and higher Hachinski Ischemic Scale score were associated with increased odds of post‐stroke dementia, whereas left‐handedness was associated with lower odds (Figure 4A).

Sex‐stratified analyses indicated differential associations across sexes (Figure 4B). Overall, neuropsychiatric and functional measures showed stronger associations in women, whereas neurological signs and cerebrovascular burden related measures were more strongly associated with post‐stroke dementia in men. Detailed sex‐specific estimates are presented in the eResults.

## 4 DISCUSSION

We developed and validated the MSDS using the NACC dataset and the AutoScore–ShapleyVIC framework. The robustness and the generalizability of the MSDS was demonstrated in an external validation using an independent dataset. Our results demonstrate that ShapleyVIC offers a robust and interpretable method for feature selection, producing a parsimonious clinical risk score with strong predictive performance for post‐stroke dementia. Notably, sex‐specific analyses revealed distinct risk factor profiles for men and women, emphasizing the importance of tailored approaches to clinical risk stratification. By complementing machine learning with epidemiological evidence, this study enhances both the robustness and clinical applicability of identified risk factors.

The risk factors identified by ShapleyVIC for the MSDS are consistent with established clinical and epidemiologic evidence. The pattern of cognitive decline, whether abrupt, stepwise, or gradual, remains a key clinical feature for predicting vascular dementia.^25^ Traumatic brain injury has been linked to an elevated long‐term risk of dementia, including both vascular cognitive impairment and Alzheimer' disease, in large cohort studies.^26^, ^27^ The Hachinski Ischemic Scale score is a strong predictive factor of post‐stroke cognitive outcomes.^21^ Abnormal neurological examination and focal neurological symptoms directly capture cerebrovascular injury, consistent with studies linking these findings to later cognitive decline,^28^, ^29^ while motor behavior changes have been associated with subcortical ischemia and subsequent dementia.^30^ Finally, marital status reflects the role of social support, with population studies showing higher dementia risk in socially isolated or unmarried individuals.^31^ Together, these findings suggest that the MSDS utilize clinically plausible and epidemiologically supported predictors, while further validation in large, population‐based cohorts will be essential to establish their prognostic value in post‐stroke dementia.

Our refined clinical score for post‐stroke dementia demonstrated that higher score corresponded to greater risk, with abrupt cognitive decline, recent brain trauma, elevated Hachinski Ischemic Scale score, abnormal neurological findings, and focal neurological symptoms contributing most strongly. These results align with our epidemiological findings identifying vascular burden and neurological deficits as key predictors of dementia risk. Conversely, marital status and gradual cognitive decline carried negligible weight, reflecting refinement through ShapleyVIC feature prioritization. This approach integrates robust epidemiologic associations with data‐driven interpretability, enhancing clinical applicability of the score for risk stratification after stroke.^32^

Beyond the overall MSDS, sex‐stratified analyses revealed distinct yet partially overlapping risk profiles. Among men, abrupt cognitive decline, traumatic brain injury, elevated Hachinski Ischemic Scale score, and primary progressive aphasia emerged as leading predictors, with further contributions from focal neurological deficits, abnormal neurological findings, and marital status. Among women, the dominant predictors included Hachinski Ischemic Scale score, focal neurological symptoms, abrupt decline, and smoking, supplemented by congestive heart failure, seizures, living situation, and clinician judgment of functional or cognitive change. Parsimony analysis selected 9 features for men versus 15 for women, suggesting greater heterogeneity of risk in women.^33^ These findings refine and extend our epidemiologic findings that vascular burden, smoking, heart failure, and neurological deficits contribute to post‐stroke dementia. Importantly, the sex‐specific MSDS models demonstrated a superior discrimination (AUC 0.84 in women; 0.85 in men) relative to the pooled model (AUC 0.81), underscoring the value of tailored prediction tools for precise, sex‐specific dementia risk stratification.^34^

Our results underscore important differences between ShapleyVIC‐ and RF‐based feature selection. Although RF identified similar leading predictors, it required 14 variables to optimize performance, compared with only 10 variables for ShapleyVIC. This reduction is clinically meaningful, as simpler models lessen data collection burden and are more feasible in rural or resource‐limited settings. Moreover, ShapleyVIC yielded narrower prediction intervals for high‐ranked predictors, reflecting greater stability compared with the instability of RF rankings in high‐dimensional data sets.^35^ By integrating Shapley values into the variable importance cloud framework, ShapleyVIC advances beyond post hoc explanation to actively guide parsimonious model construction.^17^ The finding that ShapleyVIC‐enhanced AutoScore framework outperformed both RF‐based and SHAP‐based models further supports the utility of this method for prognostic modeling. In addition to improving predictive accuracy, this approach provides a clinically interpretable score table that explicitly quantifies each variable's contribution and the associated increase in risk probability (Figure 3). Such transparency facilitates clinical decision‐making by translating complex machine learning outputs into a simple, point‐based system that can be readily understood. Moreover, although categorization of continuous variables may result in some loss of information compared with fully continuous modeling, this trade‐off was considered acceptable given the current study's emphasis on interpretability and clinical usability. Importantly, model discrimination remained robust after categorization, supporting the suitability of this approach for practical risk stratification.

Several limitations should be acknowledged. First, although the NACC is a multicenter cohort, all data were derived from a single database, limiting external generalizability. We have externally validated MSDS using the ROSMAP dataset; however, it must be noted that this cohort is also US‐based. Therefore, validation in independent, population‐based cohorts from diverse geographic and clinical settings will be necessary to confirm the robustness of the MSDS. Second, stroke subtype (ischemic vs hemorrhagic) was unspecified, precluding subtype‐specific modeling; future studies should incorporate subtype classification, as prior work suggests distinct trajectories and risk profiles after different stroke types.^36^ Third, neuroimaging biomarkers such as white matter hyperintensities, infarct volume, and cerebral microbleeds were not included, though these markers are increasingly recognized as strong predictors of cognitive outcomes^37^; integrating imaging with clinical predictors may improve discrimination. Fourth, the NACC population is older and enriched for dementia clinic participants, which may not reflect younger or community‐dwelling stroke survivors. Given evidence that stroke incidence is shifting toward younger individuals,^38^ external validation in younger and more diverse populations will be important. Fifth, women had a slightly longer mean follow‐up duration than men in NACC, which may have introduced differential opportunity for dementia ascertainment between sexes. Future studies with harmonized follow‐up intervals or time‐to‐event modeling will be important to further evaluate and mitigate this potential source of bias. Finally, translating these refinements into reliable, parsimonious, and user‐friendly models will be essential to support individualized risk stratification, guide follow‐up intensity, and reduce the burden of post‐stroke dementia across diverse populations.

In summary, we developed the MSDS using ShapleyVIC‐enhanced AutoScore, achieving a robust and interpretable prediction of post‐stroke dementia. ShapleyVIC outperformed RF‐ and SHAP‐based methods by providing stable, transparent, and parsimonious feature selection. Sex‐specific analyses revealed distinct risk profiles. By integrating machine learning with epidemiologic evidence, the MSDS represents a clinically meaningful advance, with potential to improve prognostic assessment and guide post‐stroke care.

## CONFLICT OF INTEREST STATEMENT

The authors have no conflicts to report. Author disclosures are available in the supporting information.

## CONSENT STATEMENT

Each Alzheimer's Disease Center that contributed data to the NACC employed its own protocol to obtain informed consent from participants or, when participants were unable to provide consent, from their next of kin, caregivers, or legal guardians. Written informed consent was obtained from all participants by Rush University. All participants have consented to allow secondary analysis of the de‐identified data.

## Supporting information



SUPPORTING INFORMATION

SUPPORTING INFORMATION
