# Supplementary material for: Predicting post‐stroke dementia using sex‐specific risk factors: Development of an interpretable clinical scoring tool
Source: Alzheimers Dement (Amst). 2026 Apr 27;18(2):e70336. doi: 10.1002/dad2.70336 (PMC13116076; doi:10.1002/dad2.70336)
Supplement: Supplementary file 2 — SUPPORTING INFORMATION [file DAD2-18-e70336-s002.docx]

**eMethods**

**ShapelyVIC AutoScore framework**

The ShapelyVIC AutoScore framework consists of six modules, which are introduced below:

**Module 1: Feature ranking**

Features were ranked by their relative ShapleyVIC values across all sampled models, and average ranks were calculated to generate stable ordering. Features with negligible contribution (95% prediction intervals (PIs) at or below zero) were excluded.

ShapleyVIC is designed to evaluate the contribution of variables by considering not only the single best-performing model but a set of nearly optimal models. These are typically logistic regression models whose loss is within a small tolerance (e.g., 5%) of the minimum logistic loss. This approach reduces bias in variable importance estimation and provides uncertainty quantification: For each model ***f***, the reliance of a variable ***j*** is measured using the Shapley Additive Global Importance (SAGE) value:

$$\hat{m}_{\left\{ \mathrm{rsj} \right\}(\boldsymbol{f})}= \mathrm{SAGE}_{j\left( \boldsymbol{f} \right)}$$

When multicollinearity is present (variance inflation factor >2), the absolute SAGE value is used to ensure robustness. The uncertainty of this estimate is captured by the standard error $\sigma_{\left\{ j \right\}}(\boldsymbol{f})$.

For comparison of two variables $X_{\left\{ i \right\}}$ and $X_{\left\{ j \right\}}$ ​, the difference between their ShapleyVIC values is assumed to follow a normal distribution:


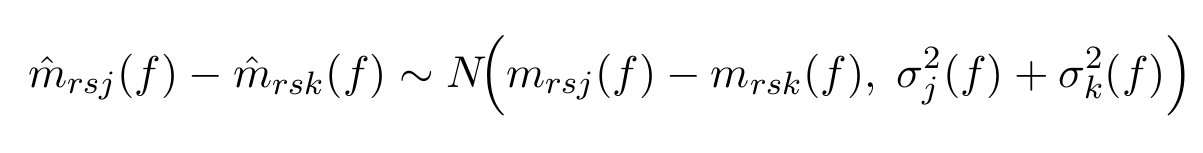


To obtain a global measure of variable importance, ShapleyVIC aggregates information across all sampled models (***M*** models in total) using a random-effects meta-analysis. This produces an overall estimate with a 95% prediction interval (PI), which explicitly quantifies the uncertainty of importance across models. Variables with intervals overlapping zero are considered non-significant and are excluded from further modeling steps. Finally, ShapleyVIC creates an ensemble ranking of variables. Each variable is ranked within each model, and the average rank across all ***M*** models provides a robust ordering:


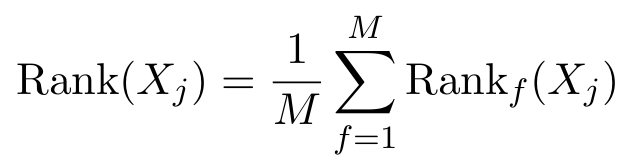


This method ensures transparent, interpretable, and statistically disciplined feature selection. It filters out noise variables, accounts for model uncertainty, and highlights predictors with consistent contributions, making it particularly well-suited for developing parsimonious and clinically interpretable risk scores.

The ShapleyVIC method comprises three steps. First, a prespecified number of nearly optimal models (250 for the current study) were sampled from a multivariable normal distribution centered on the regression coefficients of the optimal logistic regression model, where “nearly optimal” is defined by a threshold for model loss. Nearly optimal models, defined as those with loss within 5% of the minimum, yielded 250 randomly generated models for evaluation. ShapleyVIC values were estimated to be 70% of all cases to ensure stability of variable importance.

Second, the reliance of each sampled model on individual variables was quantified using the Shapley additive global importance (SAGE) method^1,2^, which measures the contribution of each predictor to model performance based on Shapley values and is closely related to the widely used SHAP approach. To account for collinearity, absolute rather than raw SAGE values were applied. Third, ShapleyVIC values were aggregated across sampled models using a random-effects meta-analysis, yielding overall estimates of variable importance and 95% PIs that quantify variability across well-performing models. Results were visualized using bar plots of mean ShapleyVIC values with 95% PIs and violin plots that illustrate the distribution of values across models.

**Optional: Original AutoScore framework with random forest feature selection**

Random forest (RF), an ensemble machine learning algorithm, has been used to identify the highest-ranking features for generating the score. RF comprises multiple tree-structured classifiers known as decision trees. Each tree is developed using a classification or regression tree method, and the final output is derived from the collective results of all trees, enhancing RF's robustness against overfitting^3^. For classification tasks, the Gini index is employed to determine the optimal split. Minimizing the Gini index reduces the probability of misclassification^4^. In the auto-score framework, the final list of features is determined by their importance rankings, alongside the parameter *m*, which represents the number of features ultimately selected. The value of *m* can be chosen on a case-by-case basis to align with clinical preferences, expert or domain knowledge, or the requirements of real-world applications.

**Module 2: Feature transformation**

All selected features undergo pre-processing and transformation. Specifically, continuous variables are converted into categorical variables to facilitate the modeling of nonlinear effects. This is a common approach to handle medical data to minimize the effects of outliers^5^. In the auto-score framework, the maximum number of categories for each variable is predefined to ensure practicality in the final risk score. For example, if the maximum number of categories is set at five, a continuous variable will be segmented into four intervals at the 0%, $25$%, $50$%, $75$%, and 100% scores of the variable's total value.

**Module 3: Score derivation**

Selected and transformed features are utilized to construct a risk score for predicting outcomes. Each category within these features is assigned to weight and given an integer point value. Multivariable logistic regression is employed to determine the weights for these scores. The formula is provided below:

$$P\left( Y=1 | X \right)=\frac{1}{1+exp(-\alpha_{0}-\alpha_{1}X_{1}-\ldots-\alpha_{n}X_{n})}$$

In this formula, $\alpha_{0}$ represents the interception, and $\alpha_{j} (1 \leq j \leq n)$ denotes the weight coefficient for each category of the feature. $X_{j} (1 \leq j \leq n)$ corresponds to each category of the features, and $Y$ is a binary outcome. The weight coefficient determines the score for each category. After fitting the data to the multivariable logistic regression model, the coefficient of $\alpha_{j}$ is obtained. The category with the lowest $\alpha_{j}$ is set as the reference category (e.g., the coefficient of the reference is set as 0). The logistic regression is then rerun with this adjusted reference to ensure no negative coefficients. After that, each $\alpha_{j}$ is divided by the lowest coefficient $\alpha_{low}$. The weighted points for each category are defined as $\alpha_{j}\left( score \right)=round(\frac{\alpha_{j}}{\alpha_{low}})$. This results in a scoring table where each category is assigned points based on its coefficient. The total score is computed by summing all points to meet specific clinical application needs.

**Module 4: Model Selection and Parameter Determination**

The model is optimized to achieve a balance between simplicity and high predictive accuracy. This is accomplished by testing different features in the training set and visualizing the relationship between model performance and complexity using a parsimony plot. The optimal number of features is identified at the point when adding additional features no longer yields a significant improvement in performance. To finalize the feature set, features are incrementally added and evaluated based on their contribution to increasing the area under the receiver of characteristic curve (AUC). If these additions necessitate adjustments, Modules 2 and 3 are re-executed to refine the model. After confirming the optimal feature set, the final list of features is determined based on their rankings from Module 1.

**Module 5: Fine-Tuning Cutoff Points**

The continuous variable cutoffs initially generated in Module 2 were refined for clinical relevance. This involved merging, rounding, or adjusting intervals to align with established medical norms and guidelines. Following these adjustments, Modules 2 and 3 were re-executed to ensure the final model maintained both robustness and clinical meaningfulness.

**Module 6: Predictive Performance Evaluation**

The performance of the developed model is evaluated using the test set, with the AUC serving as the primary evaluation metric. In addition, sensitivity and specificity are calculated to provide a comprehensive assessment of the model's performance. Where applicable, external validation is conducted using an independent dataset to further evaluate the model's robustness and generalizability.

**Epidemiology analysis**

Logistic regression models were used to estimate odds measurements, assess the associations between potential risk factors, including demographic, lifestyle, vital and sensory measurements, comorbidities, cognitive and behavioral measurements, and clinical test scores, and the odds of post-stroke cognitive impairment. Age, heart rate, and clinical test scores (CDR-SB, GDS, MMSE, and Hachinski Ischemic Score) were analyzed as continuous variables (z-scored). Education level, average number of packs smoked per day, and systolic and diastolic blood pressure were analyzed as categorical variables, while all other variables were treated as binary. All models were adjusted for age (in years), sex, education level, and race. A two-sided *P*-value<0.05 was considered statistically significant.

**Statistical analysis**

All preprocessing and statistical analyses were conducted using Python (version 3.13.5), R (version 4.3.3; RStudio version 2025.05.1+513), and Stata (version 19.0). Model development was performed with the AutoScore package, which enables streamlined construction of point-based clinical risk scores. The first 2 steps of the ShapleyVIC procedure were implemented using the Python “ShapleyVIC” package, and the final step was performed with the corresponding R package. To quantify sampling uncertainty, 95% confidence intervals were estimated from 100 bootstrap resamples. For epidemiology analyses, associations between predictors and post-stroke dementia were evaluated using odds ratios (ORs), with statistical significance defined as a 2-sided *P* < 0.05. Comparisons of AUC values between the AutoScore models and the baseline models were performed using the Wilcoxon signed-rank test (via the *rstatix* package), which is appropriate for paired AUC estimates derived from bootstrap samples^6^.

**eResult**

**1. Epidemiology findings – sex-specific**

We examined whether the associations between potential risk factors and the odds of post-stroke dementia were modified by sex. For demographic factors, being widowed, divorced, separated, or never married was significantly associated with an increased risk in males, whereas living with required assistance was significantly associated with an increased risk in females. In contrast, left-handedness was significantly associated with a decreased risk in males. For neurological conditions, females with seizures had significantly higher odds of post-stroke dementia. In addition, among cognitive and behavioral measurements, the associations for somatic complaints, emotional incontinence, clinician judgment of symptoms, and cognitive or motor changes were significantly stronger in females. Conversely, in males, the associations for pattern of cognitive impairment, focal neurological symptoms or signs, abnormal neurological exam findings, nervous system disorders (i.e. focal deficits or gait disorders), and Hachinski Ischemic Score were significantly stronger. The full statistical results are presented in **eTable 3.2**.

**eTable 1: Included features**

| Hachinski ischemic score | Pattern of cognitive decline | Focal neurological signs | Focal neurological symptoms | Focal neurological deficits |
| --- | --- | --- | --- | --- |
| Abnormal neurological exam findings | Primary progressive aphasia diagnosis | Gait disorder | Motor function change | Average number of packs smoked per day |
| Cognition status change  (memory, vision, language, judgement, attention) | Memory decline | Age | Somatic complaints | History of angioplasty/endarterectomy/stent |
| Marital status | Follow up years | Living situation | Clinical dementia rating-sum of boxes (CDR-SB) score | History of seizures |
| Thyroid disease history | Race | Normal Parkinson’s disease rating | Level of independence | Handedness |
| Active depression | Atrial fibrillation history | Urinary incontinence | Systolic blood pressure | Hypertension history |
| Hearing function | Emotional incontinence | Behavior changes | Geriatric Depression Scale (GDS) | Heart rate |
| Neuropsychiatric Inventory Questionnaire (NIQ) | History of diabetes | Diastolic blood pressure | Congestive heart failure history | Bowel incontinence |
| Type of residence | Hypercholesterole-  -mia present | History of pacemaker | Sex | Vitamin B12 deficiency history |
| History of transient ischemic attack | History of cardiac bypass surgery | Vision function | Years of education | Total years smoked cigarettes |
| History of heart attack/cardiac arrest | Mini-Mental State Examination (MMSE) score | Brain trauma with brief unconsciousness | History of traumatic brain injury | History of alcohol abuse |
| Eye movement abnormalities | History of depression | Brain incidents report | Brain trauma with extended unconsciousness |  |

For the NIQ, and GDS, only total scores were used.

**eTable 2: Performance metrics summary**

|  | **AUC** | **Sensitivity** | **Specificity** | **PPV** | **NPV** |
| --- | --- | --- | --- | --- | --- |
| **MSDS** | 0.81  (0.78-0.84) | 0.78  (0.74-0.82) | 0.77  (0.71-0.84) | 0.70  (0.69-0.72) | 0.81  (0.79-0.84) |
| **Original AutoScore** | 0.75  (0.66-0.83)  ***P* = 0.04** | 0.68  (0.63-0.69) | 0.78  (0.73-0.82) | 0.68  (0.62-0.73) | 0.75  (0.72-0.79) |
| **Logistic-RF** | 0.72  (0.68-0.75)  ***P* < 0.001** | 0.68  (0.55-0.78) | 0.72  (0.65-0.78) | 0.65  (0.56-0.70) | 0.71  (0.66-0.73) |
| **MSDS-female** | 0.84  (0.80-0.88)  ***P* = 0.02** | 0.86  (0.79-0.93) | 0.74  (0.70-0.79) | 0.72  (0.68-0.81) | 0.83  (0.79-0.86) |
| **MSDS-male** | 0.85  (0.82-0.87)  ***P* < 0.001** | 0.79  (0.72-0.83) | 0.83  (0.80-0.88) | 0.78  (0.71-0.85) | 0.80  (0.76-0.82) |

**eTable 3.1. Association between potential risk factors and odds of post-stroke cognitive impairment.**

| **Post-Stroke Cognitive Impairment Odds Ratio (OR) [95% CI], *P*-value** | | |
| --- | --- | --- |
| **Measures** | **Crude** | **Adjusted** |
| **Demographic factors** | | |
| **Age (z-scored)** | 0.87 [0.74-1.03], 0.1033 | 0.89 [0.75-1.04], 0.1461 |
| **Sex (ref: female)** | 0.88 [0.64-1.22], 0.4597 | 0.88 [0.62-1.24], 0.4562 |
| **Education (ref: ≤12 years)** |  |  |
| 13-15 years | 0.98 [0.62-1.55], 0.9443 | 0.99 [0.62-1.56], 0.9557 |
| >15 years | 1.02 [0.71-1.47], 0.9098 | 1.11 [0.76-1.63], 0.5983 |
| **Race (ref: white)** | 1.32 [0.91-1.91], 0.1389 | 1.27 [0.87-1.86], 0.2191 |
| **Marital status (ref: married)** | 1.20 [0.86-1.65], 0.2822 | 1.22 [0.84-1.77], 0.2985 |
| **Living status (ref: living with others)** | 0.91 [0.64-1.30], 0.6066 | 0.89 [0.61-1.30], 0.5511 |
| **Level of independence (ref: able to live independently)** | 1.18 [0.85-1.64], 0.3106 | 1.21 [0.87-1.69], 0.2473 |
| **Type of residence (ref: private home)** | 1.04 [0.69-1.58], 0.8439 | 1.17 [0.75-1.83], 0.4870 |
| **Left or right-handed (ref: right hand)** | **0.42 [0.21-0.83], 0.0127** | **0.40 [0.20-0.79], 0.0091** |
| **Lifestyle factors** | | |
| **Total years smoked cigarettes (ref: <1 year)** | 0.94 [0.68-1.30], 0.7087 | 0.96 [0.69-1.35], 0.8335 |
| **Average number of packages smoked per day (ref: no)** |  |  |
| 1 cigarette to less than 0.5 pack | 0.76 [0.47-1.23], 0.2681 | 0.76 [0.47-1.24], 0.2755 |
| 0.5 pack to less than 1 pack | 1.29 [0.83-2.01], 0.2537 | 1.33 [0.85-2.10], 0.2136 |
| More | 0.82 [0.52-1.31], 0.4081 | 0.87 [0.54-1.41], 0.5757 |
| **Alcohol abuse (ref: no)** | 1.11 [0.59-2.10], 0.7523 | 1.15 [0.60-2.21], 0.6749 |
| **Vital measurements** | | |
| **Systolic blood pressure (ref: normal)** |  |  |
| Elevated | 1.26 [0.73-2.17], 0.4117 | 1.26 [0.73-2.18], 0.4101 |
| High S1 | 0.94 [0.57-1.56], 0.8136 | 0.93 [0.56-1.55], 0.7789 |
| High S2 | 1.02 [0.67-1.57], 0.9139 | 1.02 [0.66-1.56], 0.9409 |
| **Diastolic blood pressure (ref: normal)** |  |  |
| Elevated | 1.18 [0.76-1.83], 0.4583 | 1.13 [0.72-1.77], 0.5920 |
| High | 0.87 [0.47-1.59], 0.6476 | 0.79 [0.43-1.47], 0.4601 |
| **Heart rate (z-scored)** | 1.11 [0.95-1.31], 0.1887 | 1.10 [0.94-1.30], 0.2412 |
| **Sensory measurements** | | |
| **Vision acuity (ref: no)** | 0.90 [0.63-1.30], 0.5788 | 0.88 [0.61-1.27], 0.4958 |
| **Hearing acuity (ref: no)** | 1.01 [0.71-1.45], 0.9573 | 0.90 [0.62-1.31], 0.5899 |
| **Cardiovascular conditions** | | |
| **Atrial fibrillation (ref: no)** | 1.24 [0.83-1.85], 0.3011 | 1.40 [0.93-2.13], 0.1107 |
| **Angioplasty endarterectomy stent (ref: no)** | **0.60 [0.36-0.99], 0.0467** | 0.62 [0.37-1.02], 0.0618 |
| **Cardiac bypass procedure (ref: no)** | 1.17 [0.70-1.96], 0.5406 | 1.24 [0.73-2.09], 0.4216 |
| **Pacemaker (ref: no)** | 0.89 [0.49-1.59], 0.6838 | 0.97 [0.54-1.76], 0.9213 |
| **Congestive heart failure (ref: no)** | 1.15 [0.65-2.04], 0.6272 | 1.22 [0.68-2.19], 0.5077 |
| **Heart attack (ref: no)** | 1.14 [0.73-1.77], 0.5600 | 1.15 [0.74-1.80], 0.5330 |
| **Transient ischemic attack (ref: no)** | 0.97 [0.66-1.41], 0.8674 | 1.02 [0.70-1.49], 0.9159 |
| **Hypertension (ref: no)** | 1.12 [0.75-1.65], 0.5839 | 1.08 [0.72-1.62], 0.7189 |
| **Neurological conditions** | | |
| **Seizures (ref: no)** | **2.64 [1.38-5.07], 0.0035** | **2.57 [1.33-4.97], 0.0052** |
| **TBI (ref: no)** | 0.95 [0.59-1.53], 0.8347 | 0.97 [0.60-1.57], 0.8993 |
| **TBI brief unconsciousness (ref: no)** | 0.97 [0.57-1.65], 0.8987 | 0.97 [0.56-1.66], 0.9044 |
| **TBI extended unconsciousness (ref: no)** | 1.20 [0.54-2.65], 0.6540 | 1.30 [0.592.90], 0.5160 |
| **Other comorbidities** | | |
| **Diabetes (ref: no)** | 1.24 [0.85-1.82], 0.2674 | 1.16 [0.77-1.75], 0.4856 |
| **Hypercholesterolemia (ref: no)** | 1.03 [0.72-1.47], 0.8861 | 0.99 [0.69-1.43], 0.9668 |
| **Thyroid disease (ref: no)** | 0.73 [0.48-1.09], 0.1241 | 0.72 [0.47-1.10], 0.1313 |
| **Incontinence – urinary (ref: no)** | 0.88 [0.62-1.25], 0.4843 | 0.93 [0.64-1.34], 0.6803 |
| **Incontinence – bowel (ref: no)** | 0.74 [0.42-1.29], 0.2877 | 0.78 [0.44-1.37], 0.3854 |
| **Active depression (ref: no)** | 1.13 [0.80-1.58], 0.4895 | 1.10 [0.78-1.55], 0.5932 |
| **Depression (ref: no)** | 1.10 [0.77-1.57], 0.6103 | 1.05 [0.73-1.52], 0.7803 |
| **Parkinson disease (ref: no)** | 0.75 [0.52-1.08], 0.1207 | 0.70 [0.48-1.01], 0.0579 |
| **Vitamin b12 deficiency (ref: no)** | 1.11 [0.67-1.85], 0.6898 | 1.15 [0.68-1.92], 0.6046 |
| **Cognitive and behavioral measurements** | | |
| **Pattern of cognitive decline (ref: no)** | **17.43 [10.81-28.10], <0.0001** | **17.87 [10.99-29.06], <0.0001** |
| **Somatic complaints (ref: no)** | **2.65 [1.46-4.79], 0.0013** | **2.64 [1.44-4.82], 0.0016** |
| **Emotional incontinence (ref: no)** | 2.20 [0.99-4.87], 0.0521 | 2.17 [0.97-4.86], 0.0591 |
| **Focal neurological symptoms (ref: no)** | **4.24 [3.01-5.98], <0.0001** | **4.21 [2.97-5.95], <0.0001** |
| **Focal neurological signs (ref: no)** | **4.24 [3.01-5.97], <0.0001** | **4.26 [3.02-6.03], <0.0001** |
| **Abnormal neurological exam findings (ref: no)** | **2.69 [1.89-3.83], <0.0001** | **2.79 [1.95-3.99], <0.0001** |
| **Nervous system disorder – focal deficits (ref: no)** | **3.17 [2.26-4.43], <0.0001** | **3.18 [2.26-4.47], <0.0001** |
| **Nervous system disorder – gait disorder (ref: no)** | **2.07 [1.43-3.02], 0.0001** | **2.08 [1.43-3.04], 0.0001** |
| **Nervous system disorder – eye movement (ref: no)** | 1.17 [0.55-2.49], 0.6791 | 1.19 [0.56-2.53], 0.6545 |
| **Clinician judgment of symptoms (ref: no)** | **2.33 [1.45-3.76], 0.0005** | **2.54 [1.56-4.13], 0.0002** |
| **Neuropsychiatric inventory (ref: no)** | 1.34 [0.95-1.91], 0.0971 | 1.36 [0.96-1.94], 0.0855 |
| **Cognition status changes (ref: no)** | **2.43 [1.51-3.90], 0.0003** | **2.64 [1.63-4.29], 0.0001** |
| **Behavior change (ref: no)** | 1.17 [0.85-1.62], 0.3417 | 1.19 [0.86-1.66], 0.2931 |
| **Motor function change (ref: no)** | **1.43 [1.03-1.99], 0.0322** | **1.52 [1.09-2.12], 0.0137** |
| **Brain incidents report (ref: no)** | 0.91 [0.33-2.51], 0.8499 | 0.90 [0.32-2.52], 0.8427 |
| **Test scores** | | |
| **CDR-SB (z-scored)** | 0.98 [0.83-1.15], 0.8013 | 0.99 [0.84-1.17], 0.9204 |
| **GDS (z-scored)** | 1.16 [0.99-1.36], 0.0613 | 1.15 [0.98-1.35], 0.0815 |
| **MMSE (z-scored)** | 1.05 [0.89-1.24], 0.5428 | 1.05 [0.89-1.25], 0.5690 |
| **Hachinski ischemic score (z-scored)** | **3.05 [2.52-3.70], <0.0001** | **3.08 [2.53-3.74], <0.0001** |

Adjusted models account for age, sex, education level, and race. Statistically significant associations (*P*<0.05) are highlighted in bold.

**eTable 3.2: Sex-stratified association between potential risk factors and odds of post-stroke cognitive impairment.**

| **Post-Stroke Cognitive Impairment Odds Ratio (OR) [95% CI], *P*-value** | | | |
| --- | --- | --- | --- |
| **Measures** | **Female** | **Male** | ***P*-interaction** |
| **Demographic factors** | | | |
| **Marital status (ref: married)** | 0.77 [0.47-1.26], 0.3042 | **2.08 [1.23-3.51], 0.0063** | **0.0066** |
| **Living status (ref: living with others)** | 0.82 [0.51-1.31], 0.4085 | 1.04 [0.55-1.94], 0.9072 | 0.5567 |
| **Level of independence (ref: able to live independently)** | **1.62 [1.02-2.58], 0.0400** | 0.89 [0.56-1.43], 0.6353 | 0.0748 |
| **Type of residence (ref: private home)** | 1.24 [0.72-2.16], 0.4369 | 1.06 [0.52-2.15], 0.8750 | 0.7155 |
| **Left or right-handed (ref: right hand)** | 0.43 [0.14-1.27], 0.1267 | **0.38 [0.15-0.93], 0.0332** | 0.8685 |
| **Lifestyle factors** | | | |
| **Total years smoked cigarettes (ref: <1 year)** | 0.94 [0.59-1.50], 0.8032 | 0.99 [0.61-1.61], 0.9653 | 0.8881 |
| **Average number of packages smoked per day (ref: no)** |  |  | 0.8951 |
| 1 cigarette to less than 0.5 pack | 0.72 [0.38-1.37], 0.3135 | 0.82 [0.39-1.74], 0.6114 |  |
| 0.5 pack to less than 1 pack | 1.55 [0.79-3.03], 0.2008 | 1.21 [0.65-2.26], 0.5514 |  |
| More | 0.78 [0.35-1.74], 0.5458 | 0.91 [0.49-1.70], 0.7738 |  |
| **Alcohol abuse (ref: no)** | 0.24 [0.03-1.94], 0.1816 | 1.62 [0.79-3.34], 0.1888 | 0.0500 |
| **Vital measurements** | | | |
| **Systolic blood pressure (ref: normal)** |  |  | 0.3489 |
| Elevated | 0.84 [0.37-1.90], 0.6822 | 1.93 [0.88-4.20], 0.0993 |  |
| High S1 | 0.73 [0.37-1.42], 0.3507 | 1.28 [0.57-2.86], 0.5537 |  |
| High S2 | 0.73 [0.41-1.29], 0.2764 | 1.54 [0.79-2.98], 0.2014 |  |
| **Diastolic blood pressure (ref: normal)** |  |  | 0.5175 |
| Elevated | 1.44 [0.78-2.63], 0.2413 | 0.86 [0.45-1.68], 0.6654 |  |
| High | 0.77 [0.33-1.79], 0.5454 | 0.82 [0.34-2.00], 0.6631 |  |
| **Heart rate (z-scored)** | 1.07 [0.85-1.34], 0.5670 | 1.14 [0.90-1.44], 0.2705 | 0.6933 |
| **Sensory measurements** | | | |
| **Vision acuity (ref: no)** | 0.93 [0.56-1.53], 0.7632 | 0.83 [0.48-1.43], 0.4995 | 0.7710 |
| **Hearing acuity (ref: no)** | 0.76 [0.45-1.30], 0.3166 | 1.05 [0.63-1.76], 0.8486 | 0.3867 |
| **Cardiovascular conditions** | | | |
| **Atrial fibrillation (ref: no)** | 1.65 [0.92-2.96], 0.0901 | 1.20 [0.67-2.15], 0.5411 | 0.4364 |
| **Angioplasty endarterectomy stent (ref: no)** | 0.72 [0.34-1.51], 0.3856 | 0.54 [0.27-1.09], 0.0850 | 0.5855 |
| **Cardiac bypass procedure (ref: no)** | 1.23 [0.48-3.13], 0.6672 | 1.24 [0.66-2.34], 0.4982 | 0.9810 |
| **Pacemaker (ref: no)** | 1.26 [0.55-2.85], 0.5843 | 0.75 [0.31-1.78], 0.5118 | 0.3896 |
| **Congestive heart failure (ref: no)** | 1.23 [0.58-2.62], 0.5841 | 1.20 [0.48-2.96], 0.6989 | 0.9578 |
| **Heart attack (ref: no)** | 1.76 [0.96-3.22], 0.0653 | 0.72 [0.36-1.42], 0.3371 | 0.0500 |
| **Transient ischemic attack (ref: no)** | 1.07 [0.64-1.81], 0.7937 | 0.97 [0.56-1.68], 0.9051 | 0.7889 |
| **Hypertension (ref: no)** | 0.79 [0.46-1.37], 0.3993 | 1.51 [0.83-2.73], 0.1781 | 0.1111 |
| **Neurological conditions** | | | |
| **Seizures (ref: no)** | **2.60 [1.10-6.15], 0.0298** | 2.52 [0.91-7.01], 0.0767 | 0.9632 |
| **TBI (ref: no)** | 0.85 [0.41-1.75], 0.6618 | 1.08 [0.57-2.05], 0.8152 | 0.6286 |
| **TBI brief unconsciousness (ref: no)** | 0.75 [0.33-1.71], 0.4916 | 1.19 [0.58-2.44], 0.6259 | 0.3981 |
| **TBI extended unconsciousness (ref: no)** | 1.75 [0.57-5.41], 0.3298 | 0.99 [0.31-3.12], 0.9843 | 0.4835 |
| **Other comorbidities** | | | |
| **Diabetes (ref: no)** | 1.14 [0.66-1.97], 0.6395 | 1.18 [0.66-2.12], 0.5786 | 0.9278 |
| **Hypercholesterolemia (ref: no)** | 1.09 [0.66-1.82], 0.7284 | 0.89 [0.53-1.50], 0.6712 | 0.5846 |
| **Thyroid disease (ref: no)** | 0.80 [0.49-1.32], 0.3852 | 0.54 [0.23-1.26], 0.1561 | 0.4259 |
| **Incontinence – urinary (ref: no)** | 1.06 [0.66-1.70], 0.7992 | 0.75 [0.42-1.35], 0.3404 | 0.3584 |
| **Incontinence – bowel (ref: no)** | 1.13 [0.55-2.30], 0.7385 | 0.45 [0.17-1.19], 0.1072 | 0.1223 |
| **Active depression (ref: no)** | 0.85 [0.53-1.36], 0.4930 | 1.48 [0.90-2.44], 0.1229 | 0.1106 |
| **Depression (ref: no)** | 0.81 [0.49-1.33], 0.4011 | 1.47 [0.86-2.51], 0.1625 | 0.1107 |
| **Parkinson disease (ref: no)** | 0.74 [0.44-1.25], 0.2606 | 0.65 [0.38-1.11], 0.1149 | 0.7239 |
| **Vitamin b12 deficiency (ref: no)** | 1.53 [0.81-2.90], 0.1900 | 0.69 [0.27-1.72], 0.4213 | 0.1482 |
| **Cognitive and behavioral measurements** | | | |
| **Pattern of cognitive decline (ref: no)** | **14.52 [7.76-27.18], <0.0001** | **23.82 [11.01-51.54], <0.0001** | 0.3247 |
| **Somatic complaints (ref: no)** | **2.79 [1.38-5.62], 0.0042** | 2.26 [0.69-7.36], 0.1770 | 0.7630 |
| **Emotional incontinence (ref: no)** | **3.67 [1.23-10.91], 0.0195** | 1.07 [0.28-4.10], 0.9180 | 0.1499 |
| **Focal neurological symptoms (ref: no)** | **3.80 [2.36-6.11], <0.0001** | **4.71 [2.84-7.81], <0.0001** | 0.5442 |
| **Focal neurological signs (ref: no)** | **4.19 [2.60-6.74], <0.0001** | **4.35 [2.63-7.19], <0.0001** | 0.9143 |
| **Physical neurological exam abnormal findings (ref: no)** | **2.50 [1.54-4.06], 0.0002** | **3.17 [1.87-5.39], <0.0001** | 0.5149 |
| **Nervous system disorder – focal deficits (ref: no)** | **2.51 [1.57-4.01], 0.0001** | **4.12 [2.51-6.77], <0.0001** | 0.1516 |
| **Nervous system disorder – gait disorder (ref: no)** | **1.88 [1.11-3.19], 0.0180** | **2.32 [1.35-3.98], 0.0023** | 0.5895 |
| **Nervous system disorder – eye movement (ref: no)** | 0.62 [0.17-2.24], 0.4674 | 1.89 [0.72-4.98], 0.1956 | 0.1614 |
| **Clinician judgment of symptoms (ref: no)** | **3.27 [1.73-6.19], 0.0003** | 1.72 [0.83-3.57], 0.1464 | 0.1977 |
| **Neuropsychiatric inventory (ref: no)** | 1.40 [0.87-2.27], 0.1666 | 1.32 [0.79-2.22], 0.2954 | 0.8646 |
| **Cognition status changes (ref: no)** | **3.44 [1.82-6.50], 0.0001** | 1.76 [0.85-3.65], 0.1301 | 0.1787 |
| **Behavior change (ref: no)** | 1.24 [0.79-1.97], 0.3513 | 1.14 [0.71-1.84], 0.5783 | 0.8040 |
| **Motor function change (ref: no)** | **1.66 [1.05-2.64], 0.0316** | 1.39 [0.86-2.23], 0.1794 | 0.5928 |
| **Brain incidents report (ref: no)** | 1.66 [0.38-7.15], 0.4995 | 0.54 [0.12-2.48], 0.4266 | 0.2902 |
| **Test scores** | | | |
| **CDR-SB (z-scored)** | 1.07 [0.86-1.34], 0.5465 | 0.90 [0.71-1.16], 0.4307 | 0.3178 |
| **GDS (z-scored)** | 1.10 [0.89-1.36], 0.3696 | 1.22 [0.96-1.54], 0.1016 | 0.5323 |
| **MMSE (z-scored)** | 0.96 [0.76-1.21], 0.7213 | 1.16 [0.91-1.48], 0.2429 | 0.2653 |
| **Hachinski ischemic score (z-scored)** | **1.54 [1.39-1.71], <0.0001** | **1.60 [1.43-1.79], <0.0001** | 0.6519 |

Adjusted models account for age, sex, education level, and race. Statistically significant associations (*P*<0.05) are highlighted in bold


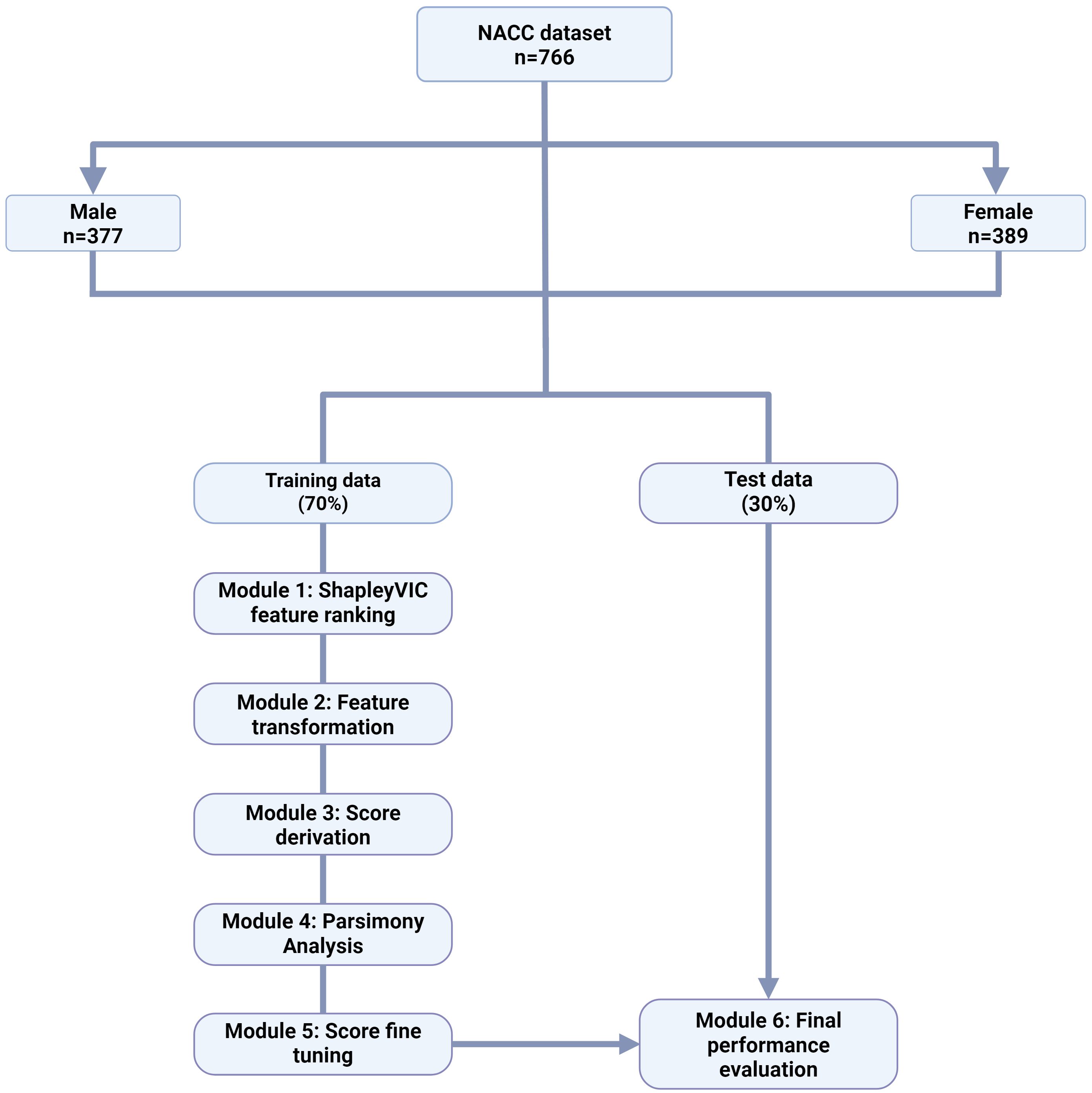


**eFigure 1. Flowchart of MSDS Development.** Two versions of the MSDS were developed using the ShapleyVIC–AutoScore framework: a general MSDS and sex-specific MSDS models.


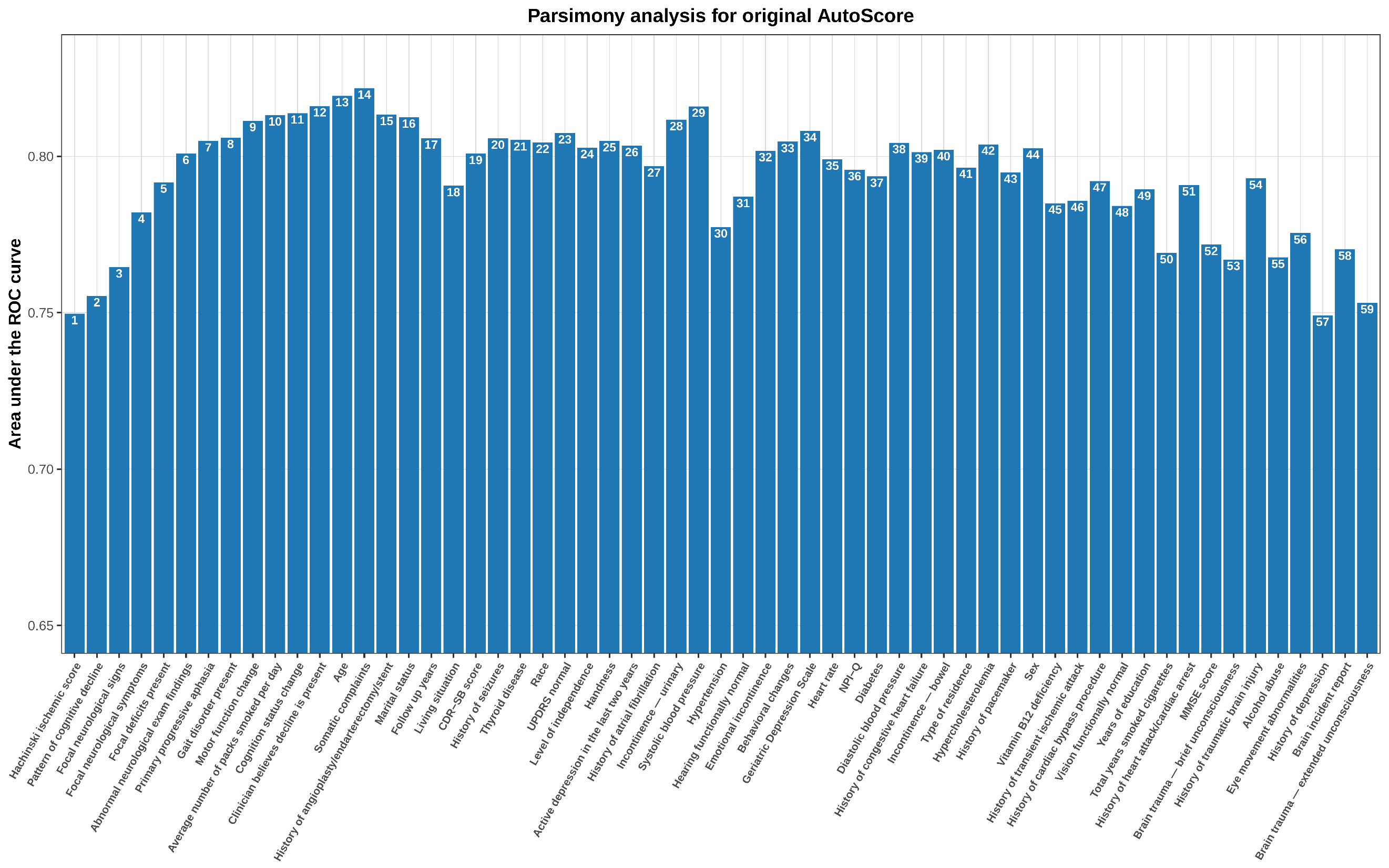


**eFigure 2: Parsimony analysis for original AutoScore.** This plot illustrates the relationship between the number of predictors included in the model and its performance, measured by the mean area under the receiver operating characteristic curve across 10-fold cross-validation. Numbers above the bars indicate the cumulative count of predictors incorporated at each step. Taller bars represent superior model performance.


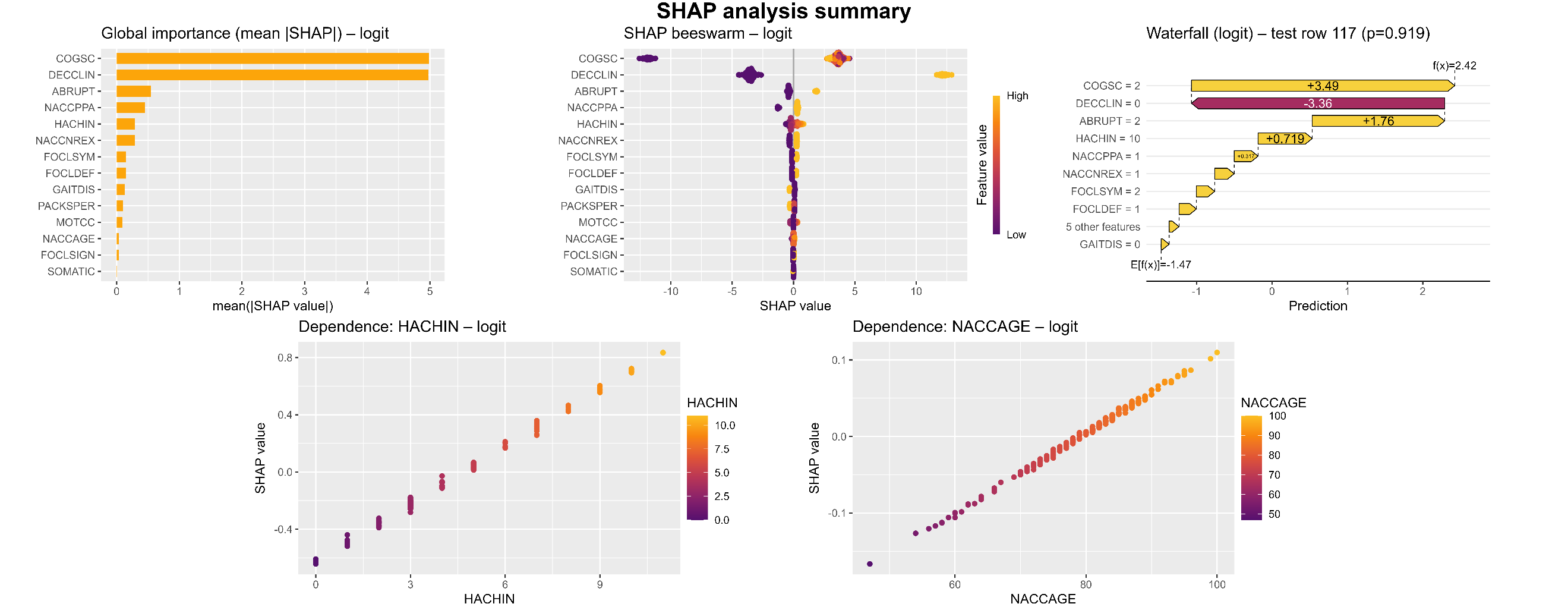


**eFigure 3: SHAP analysis summary for the Logistic–random forest model.** (A) Global importance plot shows the mean absolute SHAP values for all predictors, indicating their overall contribution to model performance. (B) SHAP beeswarm plot displays the distribution of SHAP values across individual observations, with color representing feature values. (C) Waterfall plot for an individual test case (row 117) illustrates how each predictor contributed to the final prediction probability. (D) Dependence plot for Hachinski score shows the relationship between Hachinski score and its SHAP values, with color gradient reflecting the observed feature value. (E) Dependence plot for age depicts the relationship between chronological age and SHAP values, illustrating its effect on model predictions.


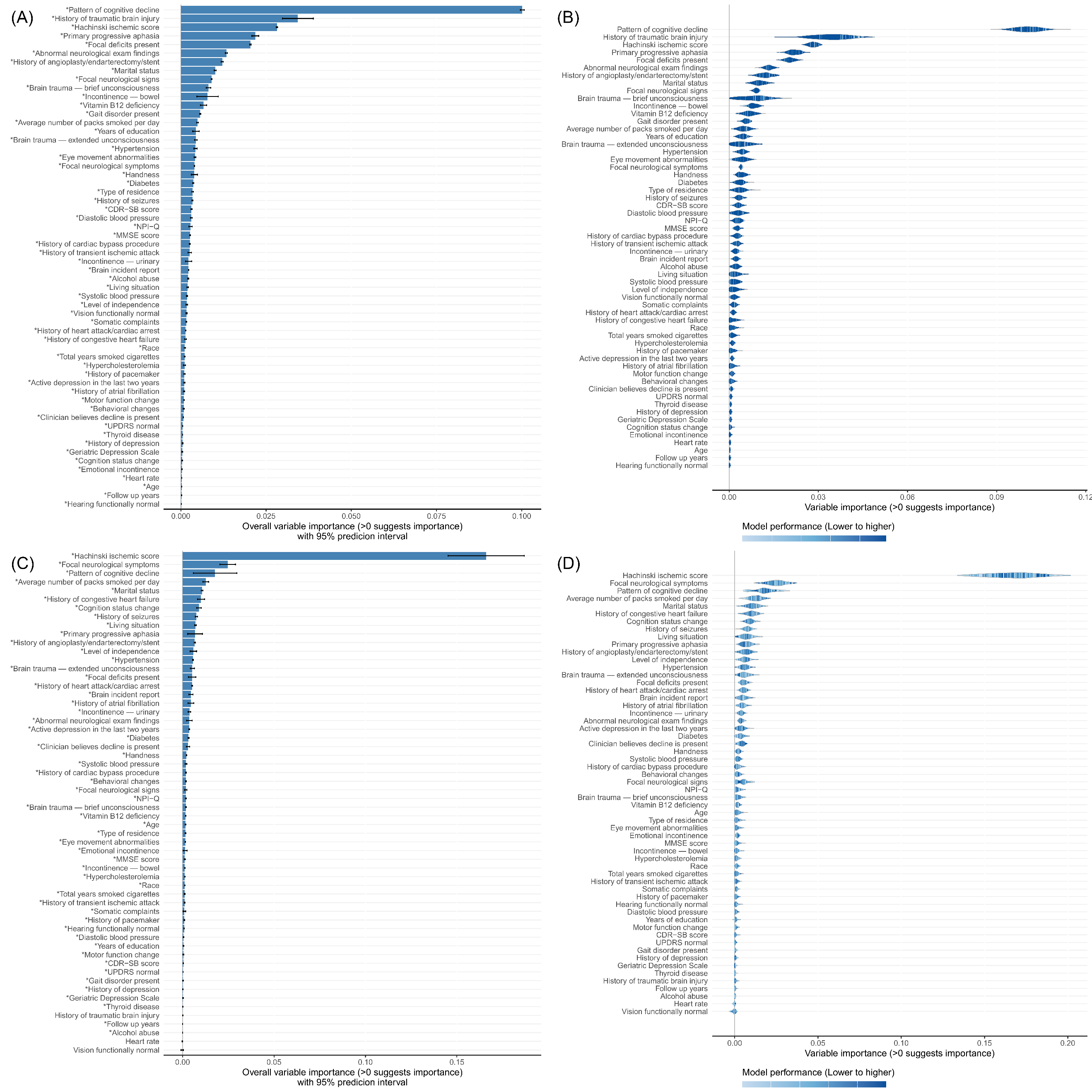


**eFigure 4. Feature Importance Ranking by Sex.** (A) Overall feature importance ranking for male participants. (B) Distribution of ShapleyVIC values for male participants. (C) Overall feature importance ranking for female participants. (D) Distribution of ShapleyVIC values for female participants.

**
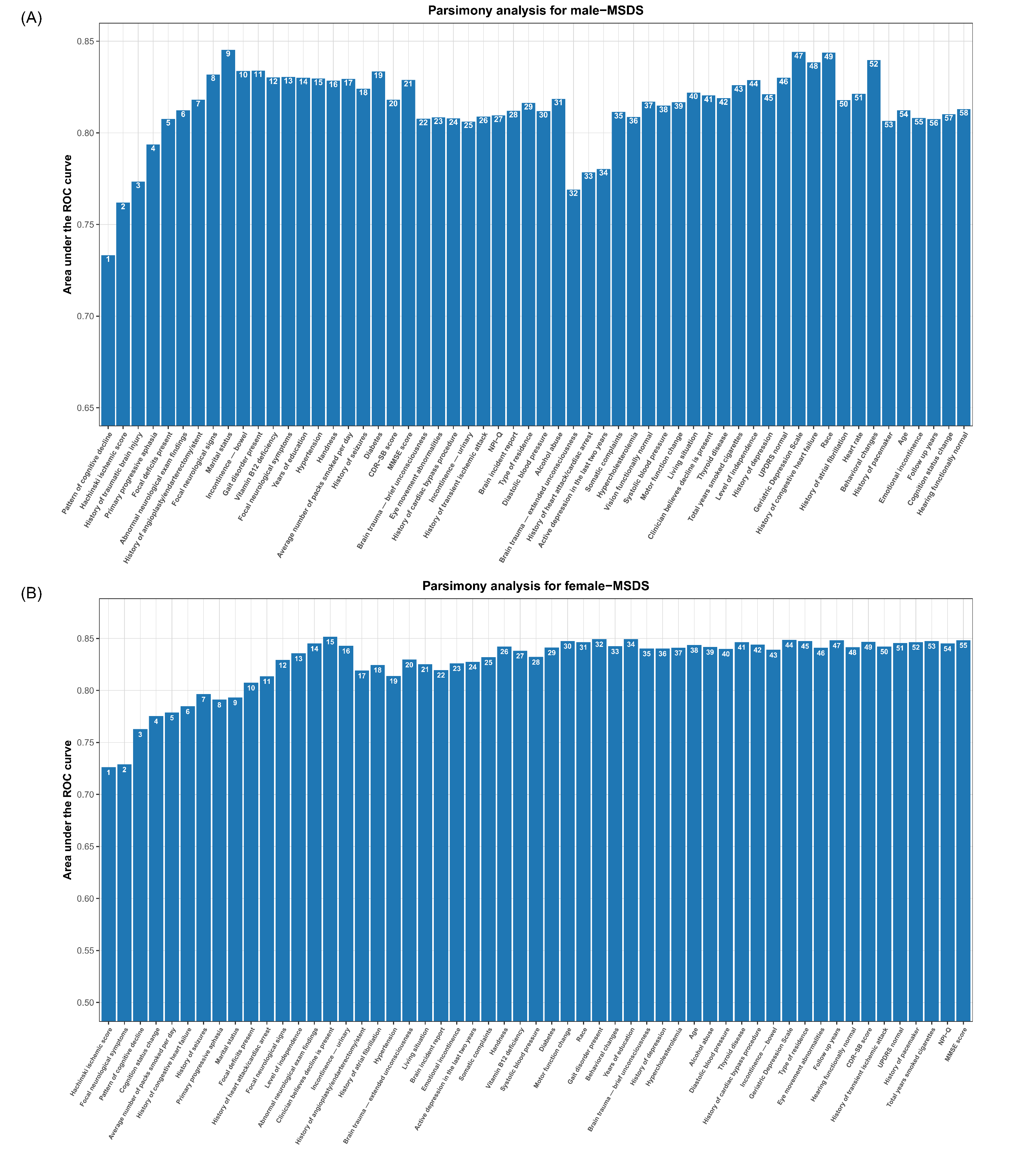
**

**eFigure 5. Parsimony Analysis by Sex.** (A) Parsimony analysis for male participants. (B) Parsimony analysis for female participants.

**Reference**

1. Covert I, Lundberg SM, Lee S-I. Understanding global feature contributions with additive importance measures. *Advances in neural information processing systems*. 2020;33:17212-17223.

2. Fryer D, Strümke I, Nguyen H. Shapley values for feature selection: The good, the bad, and the axioms. *Ieee Access*. 2021;9:144352-144360.

3. Pretorius A. *Advances in random forests with application to classification*. Stellenbosch: Stellenbosch University; 2016.

4. Kayri M, Kayri İ. The comparison of Gini and Twoing algorithms in terms of predictive ability and misclassification cost in data mining: an empirical study. *databases*. 2015;3:5.

5. Bouwmeester W, Zuithoff NP, Mallett S, et al. Reporting and methods in clinical prediction research: a systematic review. *PLoS medicine*. 2012;9(5):e1001221.

6. Crosilla F, Pillirone G. Non-parametric Statistics and Bootstrap Methods for Testing the Data Quality of a Geographic Information System. Springer Berlin Heidelberg; 1995:214-223.
